# Two Generations of “Gold Standards”: The Impact of a Decade in Hepatitis E Virus Testing Innovation on Population Seroprevalence

**DOI:** 10.4269/ajtmh.15-0159

**Published:** 2015-10-07

**Authors:** Brittany L. Kmush, Alain B. Labrique, Harry R. Dalton, Zabed B. Ahmed, John R. Ticehurst, Christopher D. Heaney, Kenrad E. Nelson, Khalequ Zaman

**Affiliations:** Department of International Health, Johns Hopkins Bloomberg School of Public Health, Baltimore, Maryland; Department of Epidemiology, Johns Hopkins Bloomberg School of Public Health, Baltimore, Maryland; European Centre for Environment and Human Health, University of Exeter, Truro, United Kingdom; Cornwall Gastrointestinal Unit, Royal Cornwall Hospital Trust, Truro, United Kingdom; Department of Microbiology, Child Health Research Foundation, Dhaka Shishu Hospital, Dhaka, Bangladesh; Johns Hopkins University School of Medicine, Baltimore, Maryland; Department of Environmental Health Sciences, Johns Hopkins Bloomberg School of Public Health, Baltimore, Maryland; Centre for Child and Adolescent Health, icddr,b, Dhaka, Bangladesh

## Abstract

Hepatitis E virus (HEV) is a global pathogen responsible for approximately 20 million infections every year in developing countries, yet remains under-recognized. In this population-based cohort study, 1,025 randomly selected participants were enrolled from Matlab, Bangladesh (2004–2005). All participants were tested for HEV antibodies and total immunoglobulin (Ig), using an in-house enzyme immunoassay developed by Walter Reed Army Institute of Research (WRAIR). In 2014, we retested the banked sera of 1,009 of those participants using the Wantai anti-HEV IgG enzyme-linked immunosorbent assay (ELISA). The WRAIR assay estimated the overall population seroprevalence as 26.6% (95% confidence interval [CI]: 24.0, 29.5), whereas the Wantai assay produced significantly higher estimated seroprevalence, 46.7% (95% CI: 43.5–49.8) (*P* < 0.001). However, the two tests give nearly identical findings in those 5 years and under (*N* = 94) with a 98% agreement between the tests. Retesting populations with modern assays is necessary to establish better population-level estimates of disease burden.

## Introduction

Hepatitis E virus (HEV) is a global pathogen responsible for approximately 20 million infections every year in developing countries alone, with an increasing recognition of high rates of autochthonous infections in developed countries as well.^1^ Despite this important burden, HEV remains an under-recognized pathogen, likely underreported as a cause of clinical illness where the pathogen is not routinely considered as part of a differential diagnosis for acute viral hepatitis. The early years of HEV research were plagued by sub-optimal commercial assays, highly variable in sensitivity and specificity.^2^,^3^ There is still no diagnostic assay approved for commercial use in the United States, with reference to specialized research laboratories required for HEV confirmation. Over the past two decades, several new, highly sensitive, and specific assays have been developed, initially in research laboratories and now, in the commercial space.

In the early 2000s, the Walter Reed Army Institute of Research (WRAIR, Silver Spring, MD) developed an in-house enzyme immunoassay (EIA) to diagnose current and past HEV infections, using an indirect approach to quantify anti-HEV total immunoglobulin (Ig) in serum.^4^ This assay uses a truncated, recombinant HEV antigen, from open reading frame (ORF)-2 of the virus, the capsid protein, expressed using the baculovirus system.^4^ This quantitative assay was extensively tested and validated by western blot and found to be more sensitive than most widely used commercial assays available at that time.^4^ A number of studies, including those performed by our research group, relied on this assay as the gold standard against which to validate commercial or other laboratory assays.^3^,^5^

Over the past few years, Beijing Wantai Pharmacy Enterprise Co., Ltd. (Beijing, China) has developed a commercially available enzyme-linked immunosorbent assay (ELISA) for detecting anti-HEV IgG. This assay also uses a segment of a recombinant ORF-2 protein, and a solid phase indirect method for quantification of anti-HEV antibodies.^6^ Several studies have validated the Wantai IgG assay against known positive and negative controls, compared with other commercially available assays and have found the Wantai assay to be the better performing assay, with a greater degree of sensitivity.^7^–^9^ However, these comparison tests have largely been completed in European populations. We had the unique opportunity to retest banked sera from a population-based serosurvey, previously examined using the WRAIR gold standard test, using this new assay, to investigate the comparability of the seroepidemiology using a newer method.

## Methods

Participants were originally selected from approximately 110,000 people included in the census of the Maternal and Child Health/Family Planning cohort of the Matlab Health Research Program of the International Center for Diarrheal Disease Research, Bangladesh (icddr,b). Matlab is a largely agrarian area in southern Bangladesh. More details of this population can be found elsewhere.^10^ A random list of 1,300 participants was generated for inclusion in a study to characterize the burden of HEV in the area over an 18-month period. More details about this parent HEV study can be found elsewhere.^11^,^12^ All individuals greater than 1 year of age were eligible for inclusion. Between 2004 and 2005 (the 1 year follow-up visit of the parent HEV study), a finger stick blood draw was collected from 1,025 consenting participants.

Shortly after the blood was drawn, the serum was tested for anti-HEV total Ig using the in-house EIA developed by the WRAIR, described above. A recommended cutoff of ≥ 20 WRAIR Units/mL was used to classify individuals as HEV antibody positive. Any remaining serum was stored at −80°C. In 2014, 1,009 of the original 1,025 samples were retested for anti-HEV IgG using the Wantai HEV-IgG ELISA according to the manufacturer's instructions. No interim freeze-thaw cycles occurred between the first and second analyses. All human subjects' research procedures were reviewed and approved by the Institutional Review Boards of icddr,b and Johns Hopkins Bloomberg School of Public Health. All subjects, or their parent or legal guardian where applicable, gave informed written consent.

Overall, and age- and gender-specific seroprevalence estimates were compared between the two assays using McNemars exact test, to account for the paired observations among individuals. Using an exact binomial distribution, 95% confidence intervals (CI) were calculated. All calculations were completed using Stata Version 11 (College Station, TX).^13^

## Results

The WRAIR assay estimated the overall population seroprevalence as 26.6% (95% CI: 24.0, 29.5), whereas the Wantai assay produced significantly higher estimated seroprevalence 46.7% (95% CI: 43.5, 49.8) (*P* < 0.001). There was a 77% agreement between the results as measured by the two assays. Using the Wantai test as the “Gold Standard,” the WRAIR assay performance was sensitivity 53.9%, specificity 97.2%, positive predictive value 94.4%, and negative predictive value 70.7%. Using the WRAIR test as the “Gold Standard,” the Wantai assay performance was sensitivity 94.4%, specificity 70.7%, positive predictive value 53.9%, and negative predictive value 97.2% (Table 1). Of the 1,009 participants with both tests completed, 444 were male (44%) and 565 (56%) were female. The WRAIR assay found 29.5% (95% CI: 25.3, 34.0) and 24.4% (95% CI: 20.9, 28.2) seroprevalence in males and females, respectively (*P* = 0.07). However, the Wantai assay found 46.6% (95% CI: 41.7, 51.2) and 46.9% (95% CI: 42.7, 51.1) seroprevalence in males and females, respectively (*P* = 0.87). The seroprevalence estimates produced from each test were significantly different in both males (*P* < 0.001) and females (*P* < 0.001).

**Figure 1.**
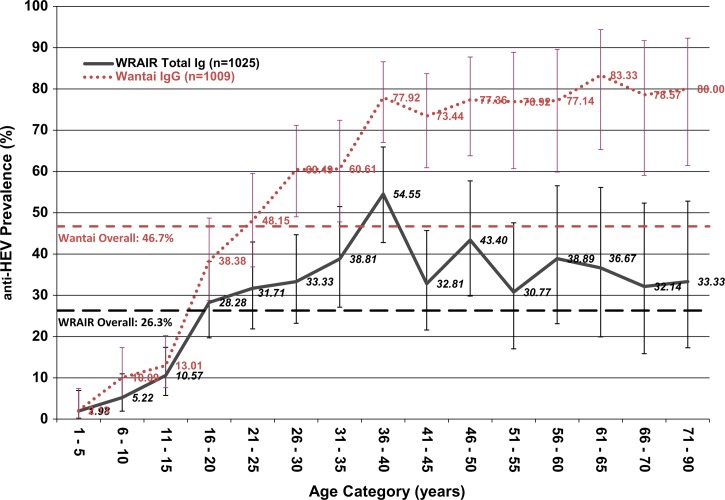
Population anti-hepatitis E virus (HEV) seroprevalence by age in Matlab, Bangladesh, a comparison of the Walter Reed Army Institute of Research (WRAIR) (*N* = 1,025) and Wantai (*N* = 1,009) assays (2004–2005). Age-stratified population anti-HEV seroprevalence using the WRAIR total immunoglobulin (Ig) assay (solid, black line) (*N* = 1,025) and the Wantai IgG assay (dotted, gray line) (*N* = 1,009) in Matlab, Bangladesh (2004–2005). Point estimates at 95% confidence intervals are displayed. Dashed lines indicate overall population seroprevalence given by the WRAIR (large, black dashes) and Wantai (small, gray dashes) assays.

The ages of the participants ranged from 2 to 89 years old; the mean age was 29.1 years (standard deviation 19.8). Both tests found seroprevalence to increase with age (Figure 1

). Young children had very low seroprevalence, with the greatest increases in seroprevalence occurring between 11 and 30 years old. The WRAIR and the Wantai assay both found similar seroprevalence among those less than 15 years old and those over 71 years old, while the seroprevalence among those 16 to 69 years differs dramatically (Figure 1, Table 2). However, it is important to note that the sample size in each age group in those over 71 years old is very small (less than 15 per age group), resulting in large confidence intervals. When those over age 70 are combined together (*N* = 30), there is a significant difference between the seroprevalence found between the WRAIR and Wantai assays (*P* < 0.001).

## Conclusions

Overall, the Wantai assay found a much higher seroprevalence of anti-HEV antibodies compared with the WRAIR assay, using the same serum. In addition, the majority of the differences between the two tests are from people initially classified by WRAIR as anti-HEV negative that Wantai classified as anti-HEV positive.

Although possible that the Wantai assay is overly sensitive and may be detecting false positives, this scenario is unlikely as the seroprevalence estimates in those 5 years and under remain very similar. The two tests give nearly identical findings in that age category (*N* = 94) with a 98% agreement between the tests. In addition, the seroprevalence estimates are not significantly different until mid-adolescence. Historically, children have had very low population seroprevalence of anti-HEV antibodies, a pattern that has been repeatedly observed across populations.^14^ It is unclear why children have low anti-HEV seroprevalence but it may be because of differences in types and amount of exposure to the virus, age-specific immune responses to HEV infection or a failure to develop a long-term immune response.^15^ The Wantai assay confirms the low seroprevalence estimate for the children tested in this study, reducing chances of false positives resulting from assay oversensitivity. Although the specificity of the Wantai IgG assay in determining distant infection has not yet been formally established, the specificity of this assay is likely to be acceptable as very low, < 5% seroprevalence rates have been found both in pediatric (this study) and adult populations in Fiji (2.2%)^16^ and New Zealand (4%).^17^

The age-specific seroprevalence pattern observed in this study, low pediatric seroprevalence followed by a steep increase in seroprevalence during adolescence, is very typical of other regions where HEV is highly endemic.^18^ In industrialized countries with low HEV endemicity, anti-HEV seroprevalence tends to be lower than in developing countries, with a low seroprevalence in children that tends to increase slowly with age, without the sharp increase during adolescence.^7^ However, because many previous studies in industrialized countries used less sensitive assays, anti-HEV seroprevalence is likely to be underestimated in low as well as high HEV-endemic areas.^7^–^9^

This study exposes the possible widespread underestimation of population seroprevalence for anti-HEV antibodies over the past two decades, even in rigorous studies that used widely accepted “gold standards” such as the WRAIR assay. However, these findings confirm the paucity of pediatric infections, which have long perplexed the HEV research community. It is likely that many population studies have significantly underestimated the burden of this disease, in both developing and developed countries. Global burden estimates developed by Rein and others in 2012 relied heavily on studies that used the WRAIR assay, potentially underestimating the global importance of this pathogen.^1^ It is imperative to retest populations with modern assays to establish better population-level estimates of disease burden as well as serologic standards to improve the quality and comparability of anti-HEV results across assays.

## Figures and Tables

**Table 1:** Comparison of the two anti-HEV immunoassays in rural Bangladesh (*N* = 1,009) (2004–2005)

Wantai IgG†	WRAIR total Ig*		Total
	Positive	Negative	
Positive	254	217	471
Negative	15	523	538
Total	269	740	1,009

HEV = hepatitis E virus; Ig = immunoglobulin; WRAIR = Walter Reed Army Institute of Research.

* WRAIR cutoff ≥ 20 Units/mL used to determine positive specimens.

† Manufacturer's signal to cutoff ratio ≥ 1 used to determine positive specimens.

**Table 2:** Comparison of WRAIR anti-HEV total Ig assay with Wantai anti-HEV IgG assay by age group in 1,009 participants from rural Bangladesh who had completed both tests (2004–2005)

Age (years)	*n*	WRAIR anti-HEV total Ig prevalence (%, 95% CI)		Wantai anti-HEV IgG prevalence (%, 95% CI)		*P* value*
1–5	94	1.98	(0.24, 6.97)	2.13	(0.26, 7.48)	1.00
6–10	109	5.22	(1.94, 11.00)	10.09	(5.15, 17.34)	0.06
11–15	123	10.57	(5.75, 17.40)	13.01	(7.62, 20.26)	0.38
16–20	99	28.28	(19.69, 38.22)	38.38	(28.78, 48.70)	0.006
21–25	81	31.71	(21.87, 42.92)	48.15	(36.90, 59.53)	0.002
26–30	81	33.33	(2.·24, 44.68)	60.49	(49.01, 71.19)	0.000
31–35	66	38.81	(27.14, 51.50)	60.61	(47.81, 72.42)	0.001
36–40	77	54.55	(42.79, 65.94)	77.92	(67.02, 86.58)	0.001
41–45	64	32.81	(21.59, 45.69)	73.44	(60.91, 83.70)	0.000
46–50	53	43.40	(29.84, 57.72)	77.36	(63.79, 87.72)	0.000
51–55	39	30.77	(17.02, 47.57)	76.92	(60.67, 88.87)	0.000
56–60	35	38.89	(23.14, 56.54)	77.14	(59.86, 89.58)	0.001
61–65	30	36.67	(19.93, 56.14)	83.33	(65.28, 94.36)	0.000
66–70	28	32.14	(15.88, 52.35)	78.57	(59.05, 91.70)	0.000
≥ 71	30	33.33	(17.29, 52.81)	80.00	(61.43, 92.29)	0.000
71–75	14	35.71	(12.76, 64.86)	71.43	(41.90, 91.61)	0.06
76–80	10	40.00	(12.16, 73.76)	90.00	(55.50, 99.75)	0.06
81–85	4	0.00	(0.00, 60.24)	75.00	(19.41, 99.37)	0.25†
86–90	2	50.00	(1.26, 98.74)	100.00	(15.81, 100.00)	1.0†

CI = confidence interval; HEV = hepatitis E virus; Ig = immunoglobulin; WRAIR = Walter Reed Army Institute of Research.

* McNemars exact test.

† Because of the small number of participants, these *P* values have limited validity.
